# Cloning of oxidosqualene cyclases from *Maytenus ilicifolia *for synthetic biology

**DOI:** 10.1186/1753-6561-8-S4-P248

**Published:** 2014-10-01

**Authors:** Cristina C Barbosa, Thaís B Alves, Sandro R Valentini, Cleslei F Zanelli, Maysa Furlan, Tatiana M Souza-Moreira

**Affiliations:** 1 Faculdade de Ciências Farmacêuticas, UNESP, Sao Paulo State University, SP, Brazil; 2 Instituto de Química, UNESP, Sao Paulo State University, SP, Brazil

## Introduction

Pentacyclic triterpenes are secondary metabolites which are promising molecules in the pharmaceutical field and food and material industries. These compounds are formed by the cyclization of oxidosqualene catalyzed by oxidosqualene cyclases (OSC) [1]. The aim of this study is to clone the OSC genes from the leaves of *Maytenus illicifolia*, a medicinal species from Brazil.

## Material and methods

For this purpose, we adopted the strategy of RT-PCR using degenerated primers obtained from sequences of known plant OSC genes. Total RNA was extracted from leaves of *M. ilicifolia *and used in the synthesis of cDNA. The PCR amplification of core fragments of OSC genes was performed with partially degenerated primers designed to anneal to highly conserved regions among OSC genes. The cloned fragments were sequenced and specific primers were designed for the rapid amplification of cDNA end (RACE) of 3' regions and for a first round amplification of 5' extension. After sequencing, another set of specific primers were generated for the RACE of 5' regions. Finally, it was possible to clone the full-length cDNA of the OSCs and confirm the predicted identity of the genes by sequencing [2].

## Results and discussion

*In silico *assembly and sequencing of the full-length cDNA covered the ORF of two main different groups of OSCs with ~2500 bp. Multiple alignment of these genes showed identity of about 50% between those two groups. Comparison of *M. ilicifolia*OSC genes with those described in the Genebank revealed that one group showed high identity (~90%) with cycloartenol synthase enzymes, while the other one showed high identity (>75%) with triterpene synthases as beta-amyrin and lupeol synthases.

## Conclusions

Cloning of OSC genes from the leaves of *M. ilicifolia *demonstrated two main groups of OSC enzymes present: the cycloartenol synthase, which is part of the plant primary metabolism and one triterpene synthase, which may be part of the secondary metabolism [3]. Future work using functional expression of these cloned genes in *Saccharomyces cerevisiae *will further characterize the oxidosqualene cyclases from *M. ilicifolia *leaves.
